# Soil water-holding capacity does not mediate aridity effects on plant functional traits in Iberian dune ecosystems

**DOI:** 10.1093/aob/mcaf184

**Published:** 2025-08-09

**Authors:** Xoaquín Moreira, Fernando T Maestre, Laura García-Velázquez, Carla Vázquez-González, Everaldo Dos Santos, Joana Serôdio, Cristina Saez-Asensio, Alexandra Rodríguez, Jorge Durán

**Affiliations:** Misión Biológica de Galicia (MBG-CSIC), Pontevedra, Galicia 36080, Spain; Environmental Sciences and Engineering, Biological and Environmental Science and Engineering Division, King Abdullah University of Science and Technology, Thuwal 23955-6900, Kingdom of Saudi Arabia; Departamento de Sistemas Físicos, Químicos y Naturales, Universidad Pablo de Olavide, Sevilla 41013, Spain; Misión Biológica de Galicia (MBG-CSIC), Pontevedra, Galicia 36080, Spain; Eixo de Recursos Naturais/Meio Ambiente, Campus Paranaguá-PR, Instituto Federal de Educação Ciência e Tecnologia do Paraná, Paranaguá CEP 83215-750 No. 453, Brazil; Centre for Functional Ecology, Associate Laboratory TERRA, Department of Life Sciences, University of Coimbra, Coimbra 3000-456, Portugal; Misión Biológica de Galicia (MBG-CSIC), Pontevedra, Galicia 36080, Spain; Misión Biológica de Galicia (MBG-CSIC), Pontevedra, Galicia 36080, Spain; Misión Biológica de Galicia (MBG-CSIC), Pontevedra, Galicia 36080, Spain

**Keywords:** *Helichrysum italicum*, Iberian Peninsula, leaf phenolics, plant growth, soil nutrients, soil water-holding capacity, specific leaf area

## Abstract

**Background and Aims:**

Aridity drives plant adaptations such as reduced stature, sclerophyllous leaves and increased phenolic production. While these patterns are well documented, the role of soil properties in modulating the impact of aridity remains understudied. Trait responses may also vary – converging, diverging, or remaining uncorrelated – across intraspecific and community levels, adding complexity to predictions of ecological responses to arid conditions.

**Methods:**

We investigated how aridity influences six plant functional traits – lateral spread, maximum height, leaf area, specific leaf area (SLA), and the concentrations of total phenolics and flavonoids – at both the species level (focusing on *Helichrysum italicum*, the dominant species across the surveyed sites) and the community level across 24 dune ecosystems along the Atlantic–Mediterranean coastline of the Iberian Peninsula. We also collected soil samples and used piecewise structural equation modelling to assess whether physico-chemical soil variables – water-holding capacity, nutrient availability, pH and organic matter content – mediate the effects of aridity on plant functional traits.

**Key Results:**

We found a significant negative relationship between aridity and both plant height and lateral spread in *H. italicum*, while leaf area, SLA, total phenolics and flavonoids were not significantly affected. At the community level, aridity was also negatively associated with plant height and lateral spread, positively associated with SLA, and showed no significant relationship with the concentrations of phenolic compounds. Importantly, water-holding capacity was strongly correlated (positively) with aridity; however, the influence of aridity on plant functional traits was not mediated by variation in this factor.

**Conclusions:**

This study demonstrates that aridity consistently influences structural plant traits across species and community levels in Iberian dune ecosystems, with largely convergent responses across organismal scales, and these patterns occur independently of key soil variables such as water-holding capacity.

## INTRODUCTION

Aridity influences ecosystem structure and dynamics by driving species to develop adaptations such as drought tolerance and efficient water use to survive in dry environments (Blanco-Sánchez *et al.*, 2022; Rodríguez-Alarcón *et al.*, 2022). As a result, aridity not only shapes species distribution but also impacts key ecological processes such as nutrient cycling, primary productivity and species interactions (Maestre *et al.*, 2022). For example, limited water availability can reduce metabolic rates, slowing plant growth and impacting herbivory and decomposition (Berdugo *et al.*, 2022; Maestre *et al.*, 2022; Sardans *et al.*, 2024). As climate change exacerbates arid conditions, understanding the ecological role of aridity becomes increasingly essential for predicting shifts in biodiversity and ecosystem functioning (Berdugo *et al.*, 2020).

In the context of plant functional traits, aridity drives a suite of adaptations that enable plants to cope with water limitations (Eldridge *et al.*, 2024; Gross *et al.*, 2024). A common strategy for coping with increasing aridity is reducing plant size. Shorter plants minimize the need for wide hydraulic conduits, which are more vulnerable to embolism under water stress (Fajardo et al., 2019). Additionally, producing smaller leaves helps reduce transpiration and overall water loss (McDonald *et al*., 2003; Yates *et al.,* 2010; Luo *et al*., 2021). Smaller leaves also reduce the surface area exposed to sunlight, mitigating the risk of water stress (Smith *et al.*, 1998). In addition to smaller leaves, plants in arid regions often develop shorter, more compact structures with limited lateral spread, minimizing exposure to harsh winds and sunlight, which helps decrease evaporation (Tumber-Dávila *et al.*, 2022; Fernández-de-Uña *et al.*, 2023). Another key adaptation involves a more efficient leaf carbon economy, often indicated by a lower specific leaf area (SLA) (León-Sánchez *et al.*, 2018; Yu *et al.*, 2022). A lower SLA reflects thicker, more robust leaves that are better at retaining moisture and storing water, thus enhancing survival in dry conditions (Vendramini *et al.*, 2002; Querejeta *et al.*, 2022). Moreover, many plants in arid environments increase the production of secondary metabolites such as phenolics (Glyphis and Puttick, 1988; Kabtni *et al.*, 2020). These compounds serve a dual function in defending against herbivores while also mitigating oxidative stress and UV radiation (Close and McArthur, 2002; Mithöfer and Boland, 2012), thereby increasing resilience to the challenges posed by aridity.

Despite significant advancements in understanding how aridity shapes plant functional traits – spanning aspects such as leaf morphology, carbon economy and secondary metabolite production (e.g. Skelton *et al*., 2015; Anderegg *et al*., 2019; Kramp *et al*., 2022; Da Sois *et al*., 2024; Gross *et al*., 2024) – critical knowledge gaps remain. One notable gap in the literature is the role of soil conditions as potential drivers of aridity effects on plant responses. Aridity does not act in isolation from other environmental variables to affect plants and their functional traits. The edaphic environment – through physical and chemical properties including water-holding capacity, nutrient availability, pH and organic matter content – plays a crucial role in shaping how plants perceive and respond to water limitation, regulating growth, resource allocation and trait expression (Delgado-Baquerizo *et al*., 2013; Eldridge *et al*., 2024). For instance, water-holding capacity determines how long moisture remains available to plants after rainfall, directly affecting water accessibility in the rooting zone (Horne and Scotter, 2016). By prolonging retention of soil moisture, high water-holding capacity can buffer plants against drought stress and modulate trait responses related to growth, biomass allocation and defence under arid conditions (Xiang *et al*., 2025). Moreover, water-holding capacity indirectly influences plant traits by stabilizing soil moisture, enhancing microbial activity, nutrient mineralization and root nutrient uptake, which improves nutrient availability (reviewed by Tariq *et al*., 2024) and may modify investment in growth, defence and resource-use efficiency (Coley *et al*., 1985; Endara and Coley, 2011). Additionally, soil pH affects nutrient solubility and microbial activity, further impacting plant physiology and performance (Viani *et al*., 2014; Barrow and Hartemink, 2023; Morrison *et al*., 2024). Gaining a deeper understanding of how soil properties mediate the impacts of aridity on plant functional traits is therefore critical for improving predictions of plant responses to ongoing environmental change.

Plant functional traits can vary significantly between intraspecific (within-species) and community (across-species) levels due to differences in ecological processes and selective pressures operating at each scale (Doudová and Douda, 2020; He *et al*., 2021). While some plant traits may exhibit similar responses to environmental stress at both levels (convergence), others may diverge or remain uncorrelated due to scale-specific mechanisms (Westerband *et al*., 2021). Intraspecific responses are typically shaped by phenotypic plasticity, genetic variability and developmental constraints (Via and Lande, 1985; Baythavong, 2011), whereas community-level patterns are driven by species turnover, environmental filtering, niche differentiation and biotic interactions such as competition (Dubart *et al*., 2022). These distinct mechanisms can lead to contrasting trait–environment relationships, highlighting the need to integrate both levels to fully understand plant functional responses to environmental change.

In this study, we investigated how aridity influences six plant functional traits across a climosequence of 24 dune ecosystems along the Atlantic–Mediterranean coastline of the Iberian Peninsula. The traits measured include lateral spread, maximum plant height and leaf area (related to leaf and whole-plant size); SLA (associated with leaf carbon economy); and concentrations of phenolic compounds – total phenolics and flavonoids – linked to resistance against abiotic and biotic stresses (Wright *et al.*, 2004; Díaz *et al.*, 2016; Gross *et al*., 2024). We initially focused on the perennial shrub *Helichrysum italicum* subsp. *picardii* (Boiss. & Reut.) Franco (Asteraceae), the dominant species present at 18 of the 24 study sites. This relatively high occurrence provided sufficient replication to explore correlations between aridity and plant functional traits within this species – an analysis not feasible for other species due to their limited distribution. To evaluate the effects of aridity more broadly, we expanded our analysis by calculating the community-weighted means (CWM) for each trait, offering a comprehensive view of the plant community’s response to aridity (Duarte *et al.*, 2018; Griffin-Nolan *et al.*, 2018). This dual approach allowed us to assess both the functional adaptations of a key species and the overall functional composition of the plant community, providing valuable insights into how aridity shapes ecosystem-level trait patterns. Two previous studies revealed variability in several soil variables across the studied climosequence (García-Velázquez *et al.*, 2020; Fernández-Alonso *et al.*, 2021). Therefore, to explore potential mechanisms, we collected soil samples to assess whether chemical and physical soil variables – water-holding capacity, nutrient availability, pH and organic matter content – mediate the influence of aridity on plant functional traits. Specifically, we address two research questions: (1) How does aridity influence plant functional traits at both the species level (*H. italicum*) and the community level, and do these effects differ between these levels? (2) Do soil chemical and physical properties mediate the relationship between aridity and plant functional traits? Overall, we aim to elucidate how soil–plant interactions shape functional trait variation across different levels of biological organization (plant species and community) and contribute to plant survival strategies under contrasting environmental conditions.

## MATERIAL AND METHODS

### Study area and climatic data

We examined 24 coastal dune ecosystems along a climosequence spanning approximately 1500 km of coastline, extending from northwest to southeast across the Iberian Peninsula (Supplementary Data Fig. S1). The study area represented a diverse climate gradient, ranging from Mediterranean with oceanic influences in the northwest to Mediterranean dry conditions in the southeast, based on the Köppen–Geiger classification system (Kottek *et al.*, 2006). To understand the climatic variability within these ecosystems, we extracted data on mean annual precipitation (MAP), mean annual temperature (MAT), annual potential evapotranspiration (PET), and aridity index (AI) for each site. These datasets were sourced from WorldCLIM 2.0 (Fick and Hijmans, 2017) and the Global Aridity and PET datasets (Trabucco and Zomer, 2019). WorldClim 2.0, based on historical climate averages from 1970 to 2000, provides high spatial resolution (approximately 1 km^2^ at 30-second resolution) and is widely recognized and utilized in ecological research (e.g. Pearse and Hipp, 2012; Weigelt *et al*., 2013). Its broad acceptance and fine-scale resolution made it an appropriate and reliable choice for maintaining consistent climatic characterization across our 24 study sites. The climatic conditions across the climosequence were highly variable, with MAP ranging from 1441 mm year^−1^ and an MAT of approximately 13.8 °C in the northwest to just 225 mm year^−1^ and an MAT of around 18 °C in the southeast. Aridity was determined based on the UNEP AI, following the equation:


Aridity=1−(AI),whereAI=MAP/PET

The aridity index ranged from −0.28 in the northwest to 0.86 in the southeast, indicating a notable shift from wetter to drier conditions along the gradient.

The dunes were in a stabilized state, which facilitated the establishment of perennial plant species. Dominant species across the study sites included *Helichrysum italicum*, found in 18 out of the 24 sites, *Ammophila arenaria*, present in 13 out of the 24 sites, and *Crucianella maritima*, also found in 13 out of the 24 sites. These stabilized dunes, fixed by shrubs, were well preserved along the climosequence.

One of the key strengths of this study’s design was the variation in climate and photosynthetic cover across the sites while keeping soil type consistent. This approach helped minimize confounding factors related to soil type, allowing for a more focused examination of how climate influences these ecosystems. Soil type was classified in all the study sites as Arenosol derived from aeolian sands (IUSS Working Group WRB, 2015). The sites exhibited a wide range of photosynthetic cover, from 20 to 100 %, and were categorized into three main groups based on climatic conditions: Humid, Mesic and Dry (Fernández-Alonso *et al.*, 2021). Additionally, each site was categorized based on local heterogeneity by distinguishing three different microsites: plant-dominated areas (under the canopy of vascular plants), cryptogam-dominated areas (mosses and lichens) and bare soil areas (lacking perennial vascular plants). The first group, ‘Humid sites’ (*n* = 9), was located in the wetter northwest, where both plant and cryptogam cover were abundant, and bare soil was absent. The second group, ‘Mesic sites’ (*n* = 4), reflected intermediate conditions in the central part of the gradient, where all three microsite types were present. The third group, ‘Dry sites’ (*n* = 11), was situated in the southeastern part of the gradient, where plant-dominated and bare soil areas were more prevalent, with cryptogam areas being less frequent. By categorizing the sites along this climosequence, we were able to analyse the interactions between climate, soil and photosynthetic cover, which contributed to a deeper understanding of how climate influences coastal dune ecosystems across different regions of the Iberian Peninsula.

### Plant sampling and measurements

In July 2016, we conducted sampling at our study sites by establishing a 30 × 30-m plot at each location, oriented parallel to the coastline. To assess biotic cover and plant community composition, we applied the line-point intercept method (Brun and Box, 1963). Four 25-m-long transects were placed within each plot, with species data recorded every 20 cm along each transect. We identified all plant species along the transect and measured both the maximum plant height and lateral spread of each individual plant. For lateral spread, two measurements were taken in perpendicular directions: north–south and east–west. The final lateral spread value for each plant was calculated as the average of these two measurements. Additionally, we collected up to ten leaves from each plant, ensuring that the leaves were evenly distributed throughout the plant’s canopy. In total, we collected leaves from 632 individual plants representing 33 species. After collection, the leaves were transported to the laboratory, where they were oven-dried for 48 h at 40 °C to standardize moisture content.

For each plant, we photographed all leaves and estimated their leaf area using ImageJ software (v.1.52a; LOCI, University of Wisconsin, USA). Additionally, we weighed all leaves to calculate SLA (cm^2^ g^−1^), providing a measure of leaf area relative to leaf mass. The leaf area and SLA measurements were averaged across the leaves to obtain a mean value for each individual plant. For 18 out of the 24 study sites, we finely ground all oven-dried leaves using liquid nitrogen and combined all plants of the same species into one sample to obtain a single value per species per site. We then extracted 20 mg of plant tissue using 70 % methanol in an ultrasonic bath for 15 min, followed by centrifugation and dilution of the methanolic extract, as described by Moreira *et al.* (2014). To determine the total phenolic content, we employed the Folin–Ciocalteu method, measuring absorbance at 740 nm using a Bio-Rad 650 microplate reader (Bio-Rad Laboratories, PA, USA) and tannic acid as a standard (Sampedro *et al.*, 2011). Additionally, to quantify the flavonoid content, we used the aluminium chloride method, measuring the absorbance of the solution at 510 nm with catechin as the standard (Zhishen *et al.*, 1999). All phenolic compound concentrations were expressed in mg g^−1^ dry weight (DW).

### Soil sampling and measurements

At each study site, we conducted stratified random sampling within three microsites: areas covered by vegetation, areas dominated by cryptogams and bare soil patches. In each microsite, we collected five soil samples from the top 10 cm of the soil profile using a 10 × 10 × 10-cm square sampler. The five samples from each microsite were pooled and homogenized to create a composite sample. To calculate the overall soil properties for each plot, we used a weighted average based on the proportional cover of each microsite (Fernández-Alonso *et al.*, 2021). Visible roots and stones were removed before sieving the samples with a 2-mm mesh. To minimize seasonal bias, soil samples were collected during the dry season (Delgado-Baquerizo *et al.*, 2016), air-dried for 1 month in the laboratory and stored in polyethylene bags until further analysis.

Soil pH was measured using a calibrated pH meter at a suspension ratio of 1:2.5 (weight:volume) in water. Soil water-holding capacity was determined using the percolation method (Harding and Ross, 1964). Soil organic matter content was estimated through loss-on-ignition at 450 °C for 4 h (Nelson and Sommers, 1996). Total soil carbon and nitrogen were analysed by dry combustion using a TruSpec CN analyser (LECO Corp., St. Joseph, MI, USA). Total phosphorus was calculated as the sum of all fractions extracted sequentially from 0.5 g of air-dried soil (García-Velázquez *et al.*, 2020), following the method of Tiessen and Moir (2006), and was determined using the malachite green method (Fernández *et al.*, 1985) in a Jupiter microplate reader (Asys Hitech GmbH, Eugendorf, Austria).

### Statistical analyses

For each study site, we calculated the CWM of all traits using the *weighted.mean* function from the *stats* package in R v.4.3.3 (R Core Team, 2024). This calculation used species-level trait values, which were weighted according to the relative abundance (i.e. frequency) of each species at each site. Subsequently, we visualized the relationships between aridity and plant functional traits for both *H. italicum* and the CWM of traits using site-level bivariate plots. We also calculated Pearson’s correlation coefficients using the *stat_cor* function from the *ggpubr* package in R (Kassambara, 2023).

To investigate the mechanisms underlying the effects of aridity on plant functional traits, we performed two piecewise structural equation models (PSEMs) (Lefcheck, 2016) using site-level data – one focusing on *H. italicum* and the other on the CWM of all traits. The objective of this analysis was to uncover associations between aridity, soil properties and leaf traits, and to determine whether bottom-up forces (i.e. soil conditions) mediate the impact of aridity on plant functional traits. To reduce model complexity and focus on the most ecologically meaningful pathways, we included in both PSEMs only those leaf traits and soil variables that showed significant correlations with aridity in our dataset. While piecewise structural equation modelling is fundamentally hypothesis-driven, it is also sensitive to overparameterization, particularly with limited sample sizes. Therefore, our variable selection strategy aimed to strike a balance between theoretical expectations and empirical support, following best practices for transparent and parsimonious structural modelling (Grace *et al*., 2012; Lefcheck, 2016). That said, we formally compared two nested models – our simplified version and a more complex one including all soil variables – by examining the difference in Fisher’s C statistic (ΔC), which follows a χ^2^ distribution with Δdf degrees of freedom (Shipley, 2013). In both the community- and species-level models, we found high *P*-values (close to 1), indicating that the more complex model did not significantly improve model fit compared to the simpler one. These results support the robustness and adequacy of our reduced models. The PSEM tested the following: (1) direct associations between aridity and soil properties, (2) direct associations between aridity and leaf traits, (3) direct associations between soil properties and leaf traits, and (4) indirect associations between aridity and plant traits via soil properties. In addition, we included direct paths from water-holding capacity to both soil nitrogen and phosphorus, as well as indirect associations between aridity and soil nutrient availability via water-holding capacity. This was based on the recognition that water-holding capacity not only reflects soil water retention capacity but also plays a critical role in mediating soil nutrient dynamics. Direct associations were represented as path coefficients, while indirect associations were calculated as multiplied path coefficients along the specified causal pathways. To implement the PSEM and obtain direct association coefficient estimates (i.e. path coefficients), we used the *psem* function from the *piecewiseSEM* package (Lefcheck, 2016). We then obtained bootstrapped indirect associations (i.e. multiplied path coefficients) and their 95 % confidence intervals using the *semEff* function from the *semEff* package (Torchiano, 2020) in R (R Core Team, 2024).

## RESULTS

**Fig. 1.**
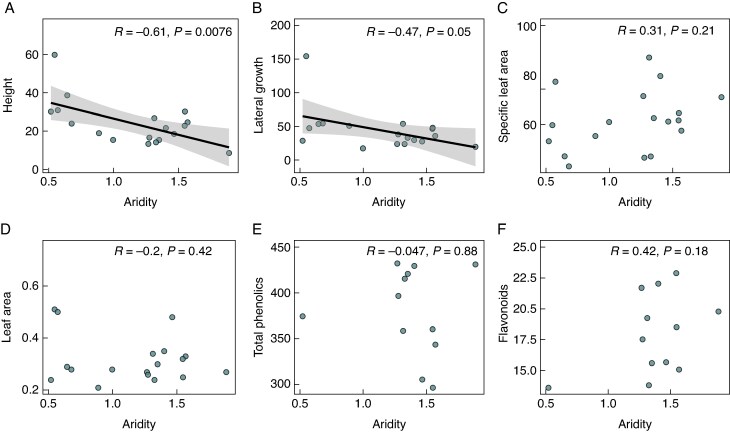
Relationships between aridity and (A) maximum plant height (cm), (B) plant lateral spread (cm), (C) leaf area (cm^2^), (D) specific leaf area (SLA, cm^2^ g^−1^), and the concentration (mg g^−1^ DW) of leaf (E) total phenolics and (F) flavonoids in *Helichrysum italicum* plants across 18 dune ecosystems located along the Atlantic–Mediterranean coastline of the Iberian Peninsula. Aridity was determined based on the UNEP Aridity Index (AI), following the equation: Aridity = 1 − (AI), where AI = MAP/PET, with MAP representing mean annual precipitation and PET representing annual potential evapotranspiration. The Pearson correlation coefficient (*R*) and *P*-values are displayed. Circles represent study sites (*N* = 18; except for phenolics and flavonoids for which *N* = 12). Black solid lines indicate significant (*P* < 0.05) relationships.

**Fig. 2.**
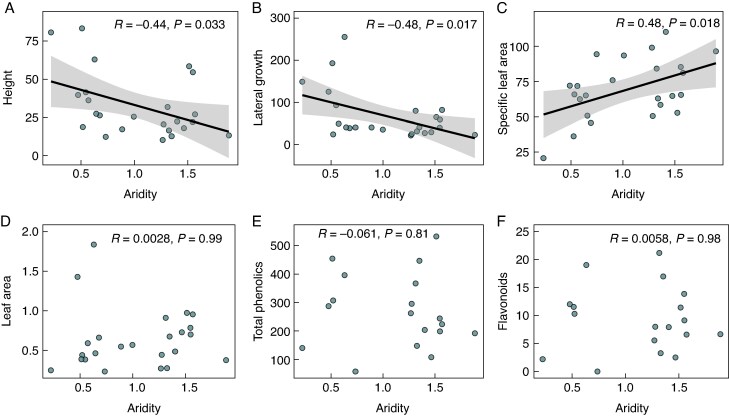
Relationships between aridity and (A) maximum plant height (cm), (B) plant lateral spread (cm), (C) leaf area (cm^2^), (D) specific leaf area (SLA, cm^2^ g^−1^), and the concentration of leaf (E) total phenolics and (F) flavonoids (mg g^−1^ DW) for the whole plant community across 24 dune ecosystems located along the Atlantic–Mediterranean coastline of the Iberian Peninsula. Aridity was determined based on the UNEP Aridity Index (AI), following the equation: Aridity = 1 − (AI), where AI = MAP/PET, with MAP representing mean annual precipitation and PET representing annual potential evapotranspiration. For each study site, the community-weighted mean of all traits was calculated using the species-level trait values, weighted by the abundance (i.e. frequency) of each species at each site. The Pearson correlation coefficient (*R*) and *P*-values are displayed. Circles represent study sites (*N* = 24; except for phenolics and flavonoids for which *N* = 18). Black solid lines indicate significant (*P* < 0.05) relationships.

We found a significant negative relationship between aridity and both plant height and lateral spread in *H. italicum*, while leaf area, SLA, total phenolics and flavonoids showed no significant associations (Fig. 1A–F). Similarly, at the community level (based on CWM values), aridity was significantly negatively related to plant height and lateral spread, positively associated with SLA, and showed no significant relationships with leaf area, total phenolics or flavonoids (Fig. 2A–F). Additionally, we found a significant positive relationship between aridity and soil nitrogen, phosphorus and water-holding capacity, while soil pH, carbon content and organic matter content showed no significant associations with aridity (Table 1).

**Table 1.** Relationships between aridity and soil variables in dune ecosystems located along the Atlantic–Mediterranean coastline of the Iberian Peninsula.

Soil variable	*R*	*P*
pH	0.33	0.121
Water-holding capacity	0.59	**0.003**
Organic matter content	0.19	0.386
Total carbon	0.19	0.361
Total nitrogen	0.51	**0**.**011**
Total phosphorus	0.55	**0**.**006**

The Pearson correlation coefficient (*R*) and *P*-values are shown (*N* = 24). Significant *P*-values (*P* < 0.05) are highlighted in bold.

**Fig. 3.**
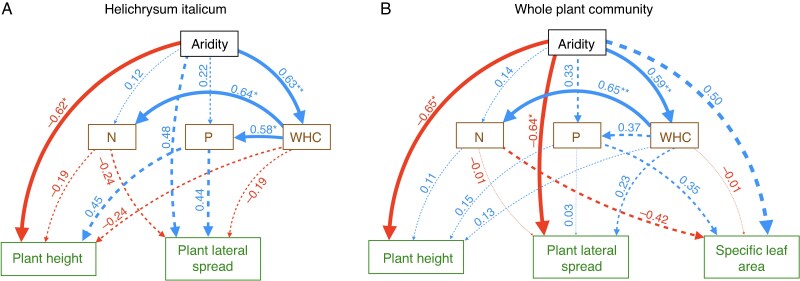
Diagram showing the path coefficients from a piecewise structural equation model testing the direct associations between aridity, soil properties [nitrogen (N), phosphorus (P) and water-holding capacity (WHC)], and plant functional traits [maximum plant height, lateral spread and specific leaf area] for (A) *Helichrysum italicum* plants and (B) the entire plant community across several dune ecosystems (*N* = 18 in A and *N* = 24 in B) along the Atlantic–Mediterranean coastline of the Iberian Peninsula. Significant path coefficients (*P < 0.05, **P < 0.01) are represented by solid arrows, while non-significant path coefficients are represented by dashed arrows. Positive relationships are shown in blue, while negative relationships are shown in red. Arrow thickness reflects the strength of the relationships between variables, as indicated by the *R*^2^ value. Indirect associations are not shown for ease of visualization. Explained variance based on marginal *R*^2^ in A: nitrogen = 0.53; phosphorus = 0.53; WHC = 0.39; plant height = 0.48; plant lateral spread = 0.33. AICc = 465.91. Explained variance based on marginal *R*^2^ in B: nitrogen = 0.27; phosphorus = 0.30; WHC = 0.34; plant height = 0.26; plant lateral spread = 0.28; specific leaf area = 0.38. AICc = 889.78.

Based on these findings, we included in both PSEMs (plant species and community level) only the plant traits and soil variables that showed significant correlations with aridity, in order to focus on the most relevant pathways. For *H. italicum*, we observed a significant positive association between aridity and water-holding capacity (Fig. 3A), as well as indirect positive associations between aridity and soil nitrogen and phosphorus via water-holding capacity ($\hat{\beta }$ = 0.315 ± 0.106 for nitrogen; $\hat{\beta }$ = 0.281 ± 0.091 for phosphorus). Aridity was also negatively associated with plant height, while no significant relationship was found with lateral spread (Fig. 3A). Additionally, none of the soil variables showed significant associations with plant functional traits, resulting in no detectable indirect effects of aridity on plant traits mediated by soil properties ($\hat{\beta }$ = −0.007 ± 0.100 for plant height; $\hat{\beta }$ = −0.004 ± 0.105 for lateral spread). At the community level, we also observed a significant positive association between aridity and water-holding capacity (Fig. 3B), along with an indirect positive association between aridity and soil nitrogen via water-holding capacity ($\hat{\beta }$ = 0.307 ± 0.080). In contrast, the corresponding indirect association with phosphorus was not statistically significant ($\hat{\beta }$ = 0.177 ± 0.104). Aridity was also significantly negatively associated with both plant height and lateral spread (Fig. 3B). However, consistent with the species-level model, soil variables were not significantly associated with any plant traits, and no indirect effects of aridity on plant traits mediated by soil properties were detected ($\hat{\beta }$ = 0.130 ± 0.094 for plant height; $\hat{\beta }$ = 0.092 ± 0.090 for lateral spread; $\hat{\beta }$ = −0.002 ± 0.113 for SLA).

## DISCUSSION

Our results indicate that aridity reduced both plant height and lateral spread at both the species level – *H. italicum* – and the community level in coastal ecosystems of the Iberian Peninsula, suggesting a consistent morphological response to water limitation across multiple organismal scales. Smaller plants with reduced lateral spread conserve water by requiring less for maintenance and growth (Li *et al.*, 2022; Tumber-Dávila *et al.*, 2022). Additionally, a smaller size and compact structure reduce the surface area through which water is lost via transpiration (Phillips and Ehleringer, 1995; Liu and Zheng, 2024), further aiding in water conservation. In arid environments, limited water availability often leads to reduced photosynthetic capacity, resulting in slower or stunted growth (reviewed by Ahluwalia *et al.*, 2021). This diminished photosynthetic capacity limits the energy available for the production of new tissues, such as leaves and stems (Lawlor and Tezara, 2009; Pinheiro and Chaves, 2011), which typically contribute to plant height and lateral spread. Moreover, in response to aridity, plants may allocate a greater proportion of their biomass below ground rather than above ground, increasing the root-to-shoot ratio (O’Brien *et al.*, 1995; Anderegg *et al.*, 2019; Tumber-Dávila *et al.*, 2022). By prioritizing root development, plants can better access and retain water from deeper soil layers, reducing the need for extensive above-ground growth that could increase water loss (Thorup-Kristensen *et al.*, 2020). These adaptive strategies enable plants to maximize water-use efficiency and improve their chances of survival in water-limited environments.

The convergence of trait responses across organizational levels in our study supports the idea that aridity acts as a strong environmental filter, selecting for similar drought-adaptive strategies within dominant species and across co-occurring species (Miao *et al*., 2025). This similarity probably arises because dominant species strongly influence community-level trait averages, and shared environmental constraints favour functionally analogous adaptations among species in these dune ecosystems (Kandlikar *et al*., 2022). However, we acknowledge contrasting findings in the literature. For example, Gross *et al*. (2024) report divergence in trait values with increasing dryness, suggesting that aridity may sometimes drive species-specific or idiosyncratic responses rather than convergence. One possible explanation for this discrepancy is that the ecological contexts and community compositions differ between studies. Our study focuses on coastal dune ecosystems where environmental filtering and limited species pools may promote convergence, while Gross *et al*. (2024) examined more heterogeneous or broader environmental gradients allowing for greater trait divergence. Additionally, differences in the dominant functional groups, species’ evolutionary histories or scales of analysis (e.g. intra- versus interspecific) could also account for these divergent patterns. Thus, while our results highlight convergence driven by strong environmental filtering, we emphasize the context-dependence of trait responses to aridity across ecosystems.

Our results showed that plants in more arid areas exhibited higher SLA at the community level. Some species may increase SLA under water-limited conditions to optimize photosynthesis and improve water-use efficiency, especially when they can effectively regulate water loss through mechanisms such as stomatal control, allowing them to maintain carbon assimilation despite reduced water availability (Li *et al*., 2022; García-Carreras *et al*., 2018). Additionally, the increase in SLA may be linked to summer deciduousness, as some species adopt a stress avoidance strategy by dropping their leaves at the onset of the dry season (Fernández-Molano *et al*., 2025). Interestingly, this positive SLA–aridity relationship was not observed in *H. italicum*, suggesting that the species may adopt a different adaptive strategy by prioritizing water conservation through lower SLA. A reduced SLA limits leaf surface area and thereby minimizes transpiration, which is crucial for survival in arid environments (Vendramini *et al*., 2002; Querejeta *et al*., 2022). *Helichrysum italicum* may further compensate for its lower SLA with other traits, such as efficient stomatal regulation, to limit water loss (Yang *et al*., 2021). In contrast, other species in the community may display greater plasticity in SLA, allowing them to respond more dynamically to aridity and enhance carbon fixation (García-Carreras *et al*., 2018). These differing strategies probably contribute to the observed increase in SLA with aridity at the community level, highlighting the diversity of plant responses to water stress (Fajardo and Siefert, 2019). It is important to note that, while focusing on a single species limits the generalizability of our species-level findings, the analysis of *H. italicum* provides a valuable case study that demonstrates how intraspecific responses can align with broader community-level patterns, offering key insights into the diverse adaptive strategies plants use in arid environments.

We found that aridity did not significantly influence phenolic compound production at the plant community level or in *H. italicum*. Several factors may explain these findings. First, the timing of our sampling may not have coincided with the peak production of phenolic compounds, as they can accumulate over a longer period or in response to specific stress events. Additionally, phenolic compounds are not always be directly linked to water availability; other environmental factors, such as herbivory, nutrient levels or soil microbial communities, can also influence their production (Sampedro *et al*., 2011; Mithöfer and Boland, 2012; Bao *et al*., 2022). These factors can vary across sites, potentially masking any clear relationship with aridity. Secondary metabolites such as phenolics are energetically costly for plants to produce, and their synthesis may be prioritized only when other stressors, such as herbivory or pathogen pressure, are more pronounced (Leitner *et al.,* 2005; Sallé *et al.*, 2005; Agrawal *et al.,* 2009). In arid environments, where water is the primary limiting factor, plants may allocate resources toward strategies that enhance water-use efficiency and survival, rather than investing in costly secondary metabolite production (He *et al.,* 2022). This trade-off between survival and chemical defence probably explains the absence of a significant correlation between aridity and phenolic or flavonoid content in our study. While phenolics play a role in plant stress responses, other environmental pressures may be more influential in shaping their production under varying aridity conditions.

Finally, we found that aridity increased soil water-holding capacity, a relatively uncommon pattern (Shi *et al*., 2024) that may result from aridity-driven changes in soil texture – such as increased fine particle accumulation or altered aggregation – that enhance moisture retention despite lower rainfall. This enhanced water-holding capacity, in turn, increased the availability of nitrogen and phosphorus by retaining moisture longer, which facilitates microbial activity and promotes nutrient mineralization, as previously reported (reviewed by Tariq *et al*., 2024). However, these soil variables did not mediate the effects of aridity on plant functional traits in either *H. italicum* or the whole plant community. Several alternative explanations are plausible. First, aridity probably exerts a more immediate and dominant influence on plant traits through direct water stress and drought tolerance mechanisms (Welles and Funk, 2020; Hartill *et al*., 2023), overriding soil nutrient effects. Second, plants in arid ecosystems often adopt conservative resource-use strategies, prioritizing survival traits over growth responses linked to nutrient availability (Zhao *et al*., 2017; Shihan *et al*., 2022). Third, aridity may exceed a threshold beyond which soil factors such as water-holding capacity have minimal influence on plant traits, making aridity itself the primary limiting factor (D’Odorico *et al*., 2013; Berdugo *et al*., 2022). Additionally, uniform soil types and inherent spatial heterogeneity in dune systems may reduce detectable soil-mediated effects at the scale of measurement (Schlesinger and Pilmanis, 1998; Huxman *et al*., 2005). Lastly, plants may exploit microsites with more favourable conditions than those captured by plot-level soil measures, decoupling average soil properties from functional trait responses. Together, these factors help explain why water-holding capacity did not act as a mediator in our study despite its positive correlation with aridity.

In conclusion, our study found that soil variables strongly correlated with aridity, such as water-holding capacity, did not mediate the effects of aridity on plant traits. This suggests that aridity itself is the primary limiting factor across the coastal Iberian Peninsula gradient. A key strength of our research is the focus on a pronounced aridity gradient with relatively stable soil conditions, minimizing confounding effects from soil type variation and associated shifts in vegetation. These findings provide novel insights into how water scarcity shapes plant adaptations and open new avenues for future research on additional ecological factors – such as herbivory and microbial communities – that may further influence plant survival strategies in arid ecosystems.

## Supplementary Material

mcaf184_Supplementary_Data
