# Supplementary material for: Soil water-holding capacity does not mediate aridity effects on plant functional traits in Iberian dune ecosystems
Source: Ann Bot. 2025 Aug 9;136(7):1565–74. doi: 10.1093/aob/mcaf184 (PMC12718070; doi:10.1093/aob/mcaf184)
Supplement: mcaf184_Supplementary_Data [file mcaf184_supplementary_data.docx]

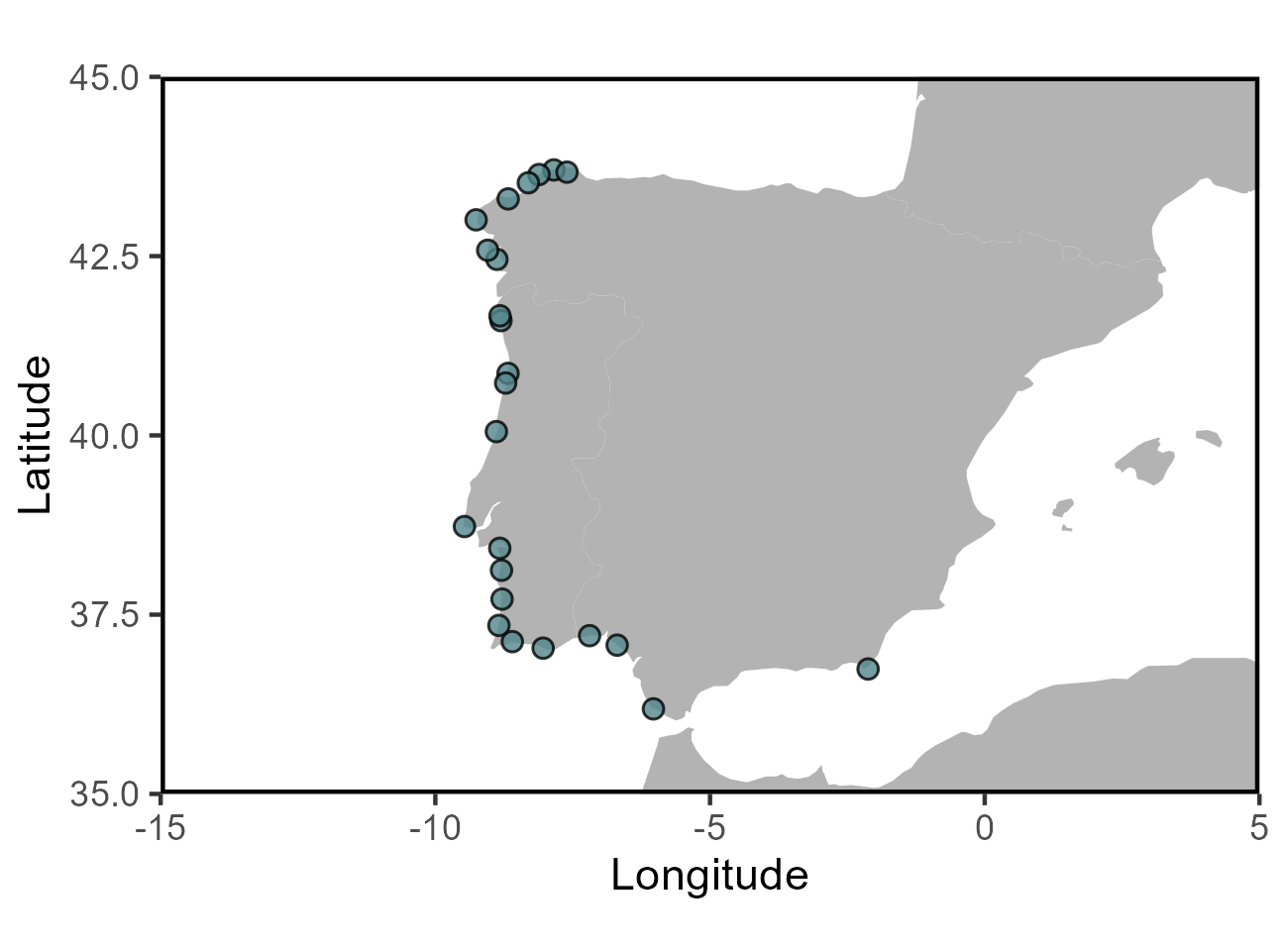


**Figure S1.** Map showing the locations of the sampling sites across 24 dune ecosystems located along the Atlantic-Mediterranean coastline of the Iberian Peninsula.
